# The Effect of Perioperative Video Interventions on Postoperative Pain Control: A Systematic Review

**DOI:** 10.1111/aas.70387

**Published:** 2026-11-01

**Authors:** Adam Danielsson, Sana Asif, Alexandra Forsberg, Miklós Lipcsey, Eva Kosek, Martin F. Bjurström

**Affiliations:** ^1^ Department of Surgical Sciences, Anesthesiology and Intensive Care Medicine Uppsala University Uppsala Sweden; ^2^ Library/ICT, Faculty of Medicine Lund University Lund Sweden; ^3^ Department of Surgical Sciences, Clinical Pain Research Uppsala University Uppsala Sweden; ^4^ Department of Clinical Neuroscience Karolinska Institutet Stockholm Sweden

## Abstract

**Introduction:**

Postoperative pain is a major cause of suffering and is linked to serious complications, including delayed wound healing, increased cardiovascular risk, delayed rehabilitation, and chronic postsurgical pain. Modifiable psychological factors influence pain outcomes, and preliminary evidence suggests video interventions can reduce preoperative anxiety and improve patient satisfaction. We hypothesized that video interventions could improve postoperative pain control. Therefore, the aim of the current systematic review was to summarize all previous research and investigate the effects of perioperative video interventions on pain levels and/or analgesic consumption.

**Methods:**

The review was registered with PROSPERO (CRD42024596389) and followed the PRISMA Guidelines. We searched PubMed, EMBASE, CINAHL, Academic Search, and the Cochrane Central register of controlled trials from inception to October 2025 for randomized controlled trials in adult surgical patients. Trials assessing any perioperative video interventions reporting on postoperative pain intensity and/or analgesic consumption were included. Data was extracted independently by two authors, and data synthesis was qualitative due to significant heterogeneity. Risk of bias was assessed using the Revised Cochrane risk‐of‐bias tool for randomized trials (RoB 2).

**Results:**

Forty‐four trials were included. Of the 42 trials assessing pain, 11 (26%) reported significantly lower pain in the intervention group. Of the 19 trials measuring analgesic consumption, eight (42%) reported significantly lower consumption in the intervention group. Only two trials demonstrated a benefit for both co‐primary outcomes. Significant improvements in secondary outcomes, including anxiety, physical function, and satisfaction, were reported in 26 trials. Most trials were judged as having a high risk of bias.

**Conclusion:**

The current systematic review suggests that perioperative video interventions may improve acute postoperative pain control, although effects are inconsistent across trials. Future research should focus on identifying patient and intervention characteristics that predict efficacy to guide clinical implementation.

**Editorial Comment:**

This trustworthy systematic review presents an overview of current research findings for application of perioperative video‐based treatments as part of managing postoperative pain. While this approach shows promise, more definitive evidence may be needed to justify more widespread implementation.

**Abbreviations**
ACT: acceptance and commitment therapy
APAIS: Amsterdam preoperative anxiety and information scale
CPSP: chronic postsurgical pain
ERAS: enhanced recovery after surgery
HHS: Harris Hip scale
NA: not assessed
NI: no information
NRS: numerical rating scale
NSAIDs: non‐steroidal anti‐inflammatory drugs
OMEQs: oral morphine equivalents
PCA: patient‐controlled analgesia
POD: postoperative day
PRISMA: preferred reporting items for systematic reviews and meta‐analyses
RoB2: revised cochrane risk‐of‐bias tool for randomized trials
SF‐36: short form (36) health survey
STAI: state–trait anxiety inventory
VAS: visual analog scale
WOMAC: The Western Ontario and McMaster Universities Arthritis Index

## 1 Introduction

Postoperative pain is a major concern, with previous studies reporting a high proportion of patients experiencing moderate to severe pain both during the first days after surgery and following hospital discharge [1, 2]. Beyond the immediate discomfort and suffering, poorly controlled postoperative pain is directly linked to multiple adverse effects, including delayed wound healing, immobilization, heightened cardiovascular risk, sleep disturbance, and delirium [3, 4, 5]. Moreover, intense acute postoperative pain significantly elevates the risk of developing chronic postsurgical pain [CPSP] [6], underscoring the critical need to enhance pain management after surgery.

Despite significant advances in multimodal analgesia, postoperative pain control remains a persistent clinical challenge [7]. Consequently, there is growing interest in targeting modifiable psychological risk factors that contribute to high postoperative pain intensity, including anxiety, depression, sleep disturbance, and pain catastrophizing [8, 9, 10]. Encouragingly, research demonstrates that even brief perioperative psychological interventions can be effective across various surgical settings [11, 12]. Furthermore, through education and non‐pharmacological treatment, Enhanced Recovery After Surgery (ERAS) protocols have decreased analgesic consumption [13].

Effective communication about surgical and postoperative expectations is a vital component of surgical care that is frequently overlooked. For example, patients undergoing Cesarean sections (C‐sections) often report feeling unprepared for postoperative pain and uncertain about recovery [14]. Preoperative video education has emerged as a promising, scalable solution to this problem, with a prior systematic review suggesting its potential [15]. Such informational videos have been shown to reduce preoperative anxiety and increase postoperative satisfaction [16]. However, the quality of publicly available content is highly variable; an analysis of 100 YouTube videos on C‐sections found that they generally failed to provide appropriate medical information [17]. This discrepancy underscores the necessity for evidence‐based video interventions and a clear synthesis of their impact on core clinical outcomes like pain and analgesic use.

We hypothesized that video interventions, as standardized, widely‐applicable, and cost‐efficient tools [18, 19], could improve postoperative pain control. This systematic review therefore aimed to provide a definitive assessment by evaluating the effects of perioperative video interventions on postoperative pain intensity and analgesic consumption in surgical patients.

## 2 Method

The current trial was conducted as a systematic review and reported according to the Preferred Reporting Items for Systematic Reviews and Meta‐Analyses (PRISMA) [20]. The protocol for this systematic review was registered with PROSPERO in October 2024 (ID: CRD42024596389).

### 2.1 Information Sources

An expert librarian (AF) planned and executed a comprehensive search across multiple electronic databases (PubMed, EMBASE, CINAHL, Academic Search and the Cochrane Central register of controlled trials [CENTRAL]) (Supplemental Document 1—available data from inception to October 2024). Additional databases beyond those listed in the PROSPERO registration (CINAHL and Academic Search) were searched to ensure all relevant trials were identified. The search strategy was updated and rerun prior to the final analysis in October 2025 to ensure the inclusion of the latest evidence. In addition to the electronic searches, we also screened the reference lists of all full‐text articles to find additional relevant sources.

### 2.2 Inclusion Criteria

Randomized controlled trials involving adult surgical patients who received video interventions‐either as a standalone treatment or as part of a multimodal approach during the pre‐, intra‐ or postoperative period were included, provided they reported outcomes related to postoperative pain and/or analgesic consumption. No restrictions were placed on the timing of the intervention relative to surgery, or on the timing of outcome assessment, as long as the intervention and outcomes were related to the surgical episode.

### 2.3 Exclusion Criteria

Articles were excluded if the intervention involved telehealth live video counseling, videos used solely for distraction, virtual reality, or videos aimed at next of kin. Furthermore, trials measuring only intraoperative pain or the tolerability of the surgery were excluded. Articles were also excluded if the full text was unavailable or if they were not published in English.

### 2.4 Study Selection

Screening of titles and abstracts of all results from the electronic search was performed manually and independently by two authors (AD and MFB). Subsequently, two authors (AD and SA) screened all relevant articles in full text before determining inclusion. Any disagreements were resolved through discussion with a senior author (MFB).

### 2.5 Data Collection

Data collection was performed independently by two review authors (AD and SA) using a spreadsheet with pre‐defined fields, which included the following information: year of publication, author, title, country where the research was performed, if pain and/or analgesic consumption was defined as the primary outcome measure, surgical population, number of participants, percentage of females, mean age, main video content, if the video was delivered as a single‐modality intervention or as part of a bundled intervention, timing of video intervention, video duration, mode of video delivery, source of video, differences in education level, preoperative pain/use of analgesics, baseline group differences in demographic or clinical factors, presence of pain and/or analgesia‐related exclusion criteria, intraoperative pain control, mode of anesthesia, postoperative pain intensity, postoperative analgesic consumption, timing of pain/analgesic assessment, other relevant outcomes, power calculation, and if analyses accounted for differences in pain at baseline.

### 2.6 Primary Outcomes

Two co‐primary outcomes were selected: (1) changes in pain intensity (e.g., pain numerical rating scale (NRS), visual analog scale (VAS) score) and (2) analgesic consumption (e.g., oral morphine equivalents [OMEQs] in mg) from baseline to end of intervention/follow‐up. The minimum clinically important difference in pain scores was defined as a relative reduction of 30%.

### 2.7 Additional Outcomes

Secondary outcomes included changes in any relevant outcomes, such as anxiety, depression, quality‐of‐life, health‐related function, physical activity, sleep measures, cognition, satisfaction, and adverse events, from baseline to end of intervention/follow‐up.

### 2.8 Subgroup Analyses

Preplanned investigation of subgroups included: target of the video intervention (e.g., pain management, anxiety, etc.), timing of video delivery (pre‐, intra‐, and/or postoperative), type of video intervention (stand‐alone vs. part of bundled intervention), type of video delivery (freely available vs. only viewed on a set number of occasions), length of video, type of surgery, and only trials with low risk of bias.

### 2.9 Risk of Bias Assessment

Study quality and risk of bias were assessed independently by two reviewers (AD and SA) for each of the co‐primary outcomes using the domain‐based Revised Cochrane risk‐of‐bias tool for randomized trials (RoB 2) [21]. Disagreements were resolved by discussion and consensus, including a third reviewer (MFB).

### 2.10 Strategy for Data Analysis

Our preplanned strategy for quantitative analysis and pooling of data is provided with the PROSPERO registration of this systematic review (ID: CRD42024596389). Unfortunately, the meta‐analysis of the primary outcomes was deemed inappropriate due to significant methodological heterogeneity among the included trials. Hence, the analysis was limited to a comprehensive qualitative assessment.

Among the pre‐planned subgroup analyses the target of video intervention was divided into videos having an informational focus, for example, “this is how the procedure is performed” or having an educational focus, which could include “these are ways to manage the postoperative pain”. The included videos were categorized by duration as short (0–6 min), medium (6–16 min), and long (≥ 16 min); the 6‐min threshold was selected to group videos slightly over 5 min that aligned better with the short duration content.

Type of surgery was classified as either major or minor. Major surgeries included, for example, abdominal surgery, total hip or knee arthroplasty, and C‐sections, while minor procedures included, for example, gastroscopy, urodynamics testing, and office hysteroscopy. Timing of the outcome assessment was categorized as acute (first 7 days postop), subacute (1–4 weeks postop) or long‐term (> 4 weeks postop); trials could qualify for more than one categorization.

We also examined several other characteristics: whether the intervention was a standalone video or part of a bundled approach (e.g., supplemented with brochures, handouts, or patient‐controlled analgesia (PCA) device practice); the possibility for participants to view the video more than once; if the trials used pain and/or analgesia consumption as their primary outcome; the mention of professional expertise in the video's production; study population size; and the percentage of female participants.

Certainty of evidence was not formally assessed using the Grading of Recommendations, Assessment, Development and Evaluations (GRADE) approach. Heterogeneity of outcome measures across trials precluded pooling, and data were synthesized narratively without a formal certainty rating [22].

The risk of bias summary figure was generated with the robvis web app [23].

## 3 Results

The first search (October 2024) yielded a total of 38 trials [24, 25, 26, 27, 28, 29, 30, 31, 32, 33, 34, 35, 36, 37, 38, 39, 40, 41, 42, 43, 44, 45, 46, 47, 48, 49, 50, 51, 52, 53, 54, 55, 56, 57, 58, 59, 60, 61]; the second search, performed in October 2025, yielded an additional seven trials [62, 63, 64, 65, 66, 67, 68], bringing the total number to 45. Since two of the included trials, Syed 2018 [58] and Cheesman 2020 [32], are based on the same cohort of patients (one focusing on the acute postoperative phase, the other reporting on long‐term follow‐up results), findings were merged and considered to originate from one trial, Syed 2018 [58], except for the risk of bias assessment, which was performed for both in order to find any potentially confounding factors. Hence, 44 trials were included in the final analysis (Figure 1).

**FIGURE 1:**
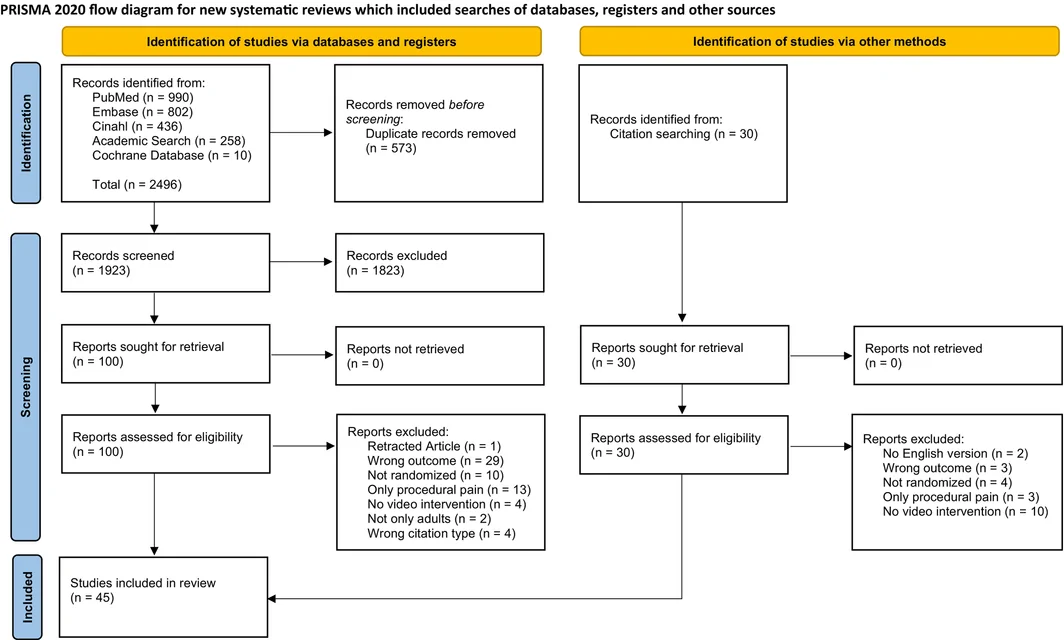
Preferred reporting items for systematic review and meta‐analyses (PRISMA) flow chart depicting study selection.

### 3.1 Characteristics of Included Trials

Basic characteristics of the included trials are shown in Table 1. Publication years ranged from 1993 to 2025, and trial cohort sizes ranged from 31 to 957 participants. The measurement instruments for pain and analgesia consumption, as well as descriptions of the trials´ analgesia regimens, are detailed in Supplemental Table 1. Among the 44 included trials, 42 measured postoperative pain levels [24, 25, 26, 27, 28, 29, 30, 31, 33, 34, 35, 36, 37, 38, 39, 40, 41, 42, 43, 44, 45, 46, 47, 48, 49, 50, 51, 52, 53, 54, 55, 56, 57, 58, 59, 60, 61, 63, 64, 65, 67, 68], while 19 measured analgesia consumption [26, 29, 34, 37, 41, 42, 45, 48, 50, 52, 53, 54, 57, 58, 59, 60, 62, 66, 68]. Pain was commonly measured using NRS or VAS, while analgesia consumption was measured by pill count or OMEQs. Seventeen of the 44 included trials examined the effect of a perioperative video intervention on both pain levels and analgesia consumption [26, 29, 34, 37, 41, 42, 45, 48, 50, 52, 53, 54, 57, 58, 59, 60, 68] while 25 trials examined only pain levels [24, 25, 27, 28, 30, 31, 33, 35, 36, 38, 39, 40, 43, 44, 46, 47, 49, 51, 55, 56, 61, 63, 64, 65, 67], and the remaining two trials examined only analgesia consumption [62, 66].

**TABLE 1:** Basic study characteristics and information about the intervention video.

First author (Year)	Surgical population	Sample size	Female (%)	Age (years, mean (range))	Main intervention video content	Study groups
Akcelik (2023) [24]	Lumbar disc herniation	60	51.6	(19–77)	Body mechanics education	Video education vs. brochure vs. standard care
Arun (2024) [25]	Cesarean section	64	100	NI	Information and instructions regarding spinal anesthesia	Video education and handout vs. standard care
Bloom (2021) [26]	Arthroscopic rotator cuff repair	130	38.4	57.5	Opioid education	Opioid education vs. standard care
Bozoghlian (2025) [62]	Primary shoulder arthroplasty	127	40.9	68 (48–87)	Safe opioid consumption, alternative pain relief and opioid disposal	Video, method for disposal and brochure vs. standard care
Çallıoğlu (2023) [27]	Office hysteroscopy	134	100	42	Information about the procedure	Informational video vs. standard care
Campagna (2020) [28]	Major abdominal surgery	160	58.1	66.3	Information on the pre‐, peri and post‐operative period	Video information vs. standard care
Carender (2022) [29]	Total hip or knee arthroplasty	183	60.6	58.6	Opioid education	Video education + ACT‐therapy via text‐message vs. video education vs. standard care
Cetinkaya Eren (2022) [30]	Total hip replacement	31	67.7	(25–70)	Information about the surgery and rehabilitation	Video‐assisted discharge information vs. standard care
Che (2020) [31]	Cesarean section	121	100	29.2	Information on complete process of anesthesia for C‐section	Video information vs. standard care
Cooke (2015) [63]	Hip and knee replacement	82	63.4	67 (36–86)	Self‐efficacy based education	Educational video vs. standard care
Darnall (2019) [33]	Breast cancer	68	100	51.2	Pain management	Pain psychoeducational video, personalized plan for surgical success and audio for relaxation vs. digital text education
Doering (2000) [34]	Hip replacement	100	38	59.6	The complete course of treatment for a real patient	Preparation video vs. standard care
Feng (2025) [64]	Lung cancer surgery	133	45	52.7	Education regarding wound care, pain relief, lifestyle adjustments and more	Booklet and access to videos vs. standard care
Goree (2021) [35]	Mixed ambulatory surgery	103	61	45.5	Information about opioids	Opioid information video vs. standard care
Hawkins (1993) [36]	Gynecologic and abdominal	60	NI	(20–70)	Information about pain control, encouragement to ask for pain relief and information on different types of analgesia	Control group with standard practice, neutral group with standard practice and videotape with information about the hospital, pain group with videotape regarding how to best deal with pain
Ihedioha (2013) [37]	Elective colorectal surgery	60	31.6	64.5	Preoperative assessment, recovery and discharge advice	Video education vs. standard care
Işıklı (2023) [38]	Implantable port catheter	84	45.2	61.6	Information about the procedure including before/after	Video information vs. standard care
Kandemir (2025) [65]	Amniocentesis	100	100	35.5	Information about amniocentesis	Video vs. standard care
Karalar (2023) [39]	Flexible Ureteroscopic Lithotripsy	60	46.7	44.9	Detailed information about ureteroscopy	Video information vs. standard care
Karunagaran (2016) [40]	Gastroscopy	72	31.9	NI	Education about the procedure	Video information vs. standard care
Knoerl (1999) [41]	General, orthopedic, neuro, gynecology, urogynecology, plastic and ENT	76	82.9	45.2	Instructional video regarding PCA use	Preoperative instructional video and practice of using PCA device vs. standard care
Lubitz (2024) [66]	Arthroscopic partial meniscectomy	166	48.2	53	Opioid education	Access to website with video and informational links vs. standard care
Maheu (2024) [42]	Total Knee Arthroplasty	93	51.6	65.2	Information and risk with opioid use; nonopioid pain‐relief strategies	Opioid information video vs. standard care
Mantica (2024) [43]	Ureteroscopy	166	NI	57.5	Explanation of the surgery	Educational video vs. standard care
Marom (2024) [44]	Amniocentesis	100	100	34.8	Education regarding the procedure	Educational video vs. standard care
Martorella (2012) [45]	Cardiac surgery	52	21	64 (41–85)	Patient‐tailored videos for post‐op pain management	Virtual program with patient tailored videos for post‐op pain management vs. standard care
McDonald (2005) [46]	Single total knee replacement	38	68.4	73.9 (65–82)	Pain communication skills	Pain communication skills video, pain information video and written material vs. pain information video and written material
McDonald (2004) [47]	Total knee arthroplasty	41	63.4	71.8	Pain communication Pain management	Pain communication video and pain management video with handout vs. pain management video with handout vs. standard care
Mokhtari (2021) [48]	Cesarean section	48	100	29.9	Pain management and pain education	Video education vs. standard care
Ozden (2024) [49]	Urodynamics	80	50	48.5 (18–64)	Education regarding the procedure	Four study groups: Educational brochure, Video education, Educational brochure with video education and a control group with standard care.
Paskey (2021) [50]	Lower extremity orthopedic surgery	107	49	47.8 (18–82)	Opioid counseling	Opioid counseling vs. standard care
Peker (2020) [51]	Trochanteric femoral fracture	56	60.7	75	Breathing and coughing exercises Postoperative knee and foot exercises	More extensive education at the hospital in combination with a video CD and educational booklet vs. standard care
Pierściński (2007) [52]	Primary inguinal hernia	31	0	55.4 (41–73)	The complete course of treatment for a real patient	Educational video vs. standard care
Rhon (2022) [53]	Spine surgery	120	33.3	45.9	Opioid education	Opioid video education vs. standard care
Sheetz (2024) [54]	Total Knee Arthroplasty	42	57	69.1 (48–80)	Opioid information and pain management	Video education with handout vs. in‐person education with handout vs. webinar
Soeters (2018) [55]	Unilateral knee‐ or hip arthroplasty	126	64	61 (37–85)	Education regarding the surgery and rehabilitation	Education from physical therapist and access to educational videos and pictures vs. standard care
Solomon (2016) [56]	Urodynamics	98	100	55.8	Education about urodynamics	Video information vs. music during procedure vs. standard care
Stepan (2021) [57]	Ambulatory hand surgery	191	45.5	60 (19–84)	Pain management education, including opioid counseling	Video information with summary on laminated card vs. standard care
Strom (2019) [67]	Lumbar spine fusion	99	65	54 (29–79)	Information about the process of the surgery	Access to website with animated information, support group and diary vs. standard care
Syed (2018) [58]	Arthroscopic rotator cuff repair	134	34	58.6	Opioid education	Opioid video education with handout vs. standard care
Turan (2025) [68]	Hip arthroplasty and laparoscopic‐assisted abdominal surgery	957	40.1	60.4	Pain expectations, goal of pain treatment and non‐opioid alternatives	Educational video vs. general video about surgery
Uysal (2023) [59]	Hysterectomy	96	100	47.4	PCA information	Video information vs. brochure information vs. verbal information
Weekes (2021) [60]	Arthroscopic rotator cuff repair	146	45	60	Instructions for breathing technique for relaxation	Video instructions with pamphlet vs. standard care
Zieren (2007) [61]	Elective groin hernia surgery	100	6	NI	Information about the entire surgical process/recovery	Video information vs. standard care

Abbreviations: ACT, acceptance and commitment therapy; NI, no information; PCA, patient‐controlled analgesia.

### 3.2 Risk of Bias Assessment

Risk of bias assessment for pain intensity is presented in Figure 2. Two of the included trials had a low risk of bias [62, 68], one with some concerns [36] while the remaining 42 had a high risk of bias [24, 25, 26, 27, 28, 29, 30, 31, 32, 33, 34, 35, 37, 38, 39, 40, 41, 42, 43, 44, 45, 46, 47, 48, 49, 50, 51, 52, 53, 54, 55, 56, 57, 58, 59, 60, 61, 63, 64, 65, 66, 67]. The most common reasons for a high risk of bias were lack of allocation concealment, lack of blinding combined with the use of patient‐reported outcomes, and missing data likely influenced by knowledge of the intervention. The RoB 2 assessment for analgesic consumption is presented in Supplemental Figure 1, as the domain judgments largely paralleled those for pain intensity. The answers for each signaling question are presented in Supplemental Tables 2 and 3.

**FIGURE 2:**
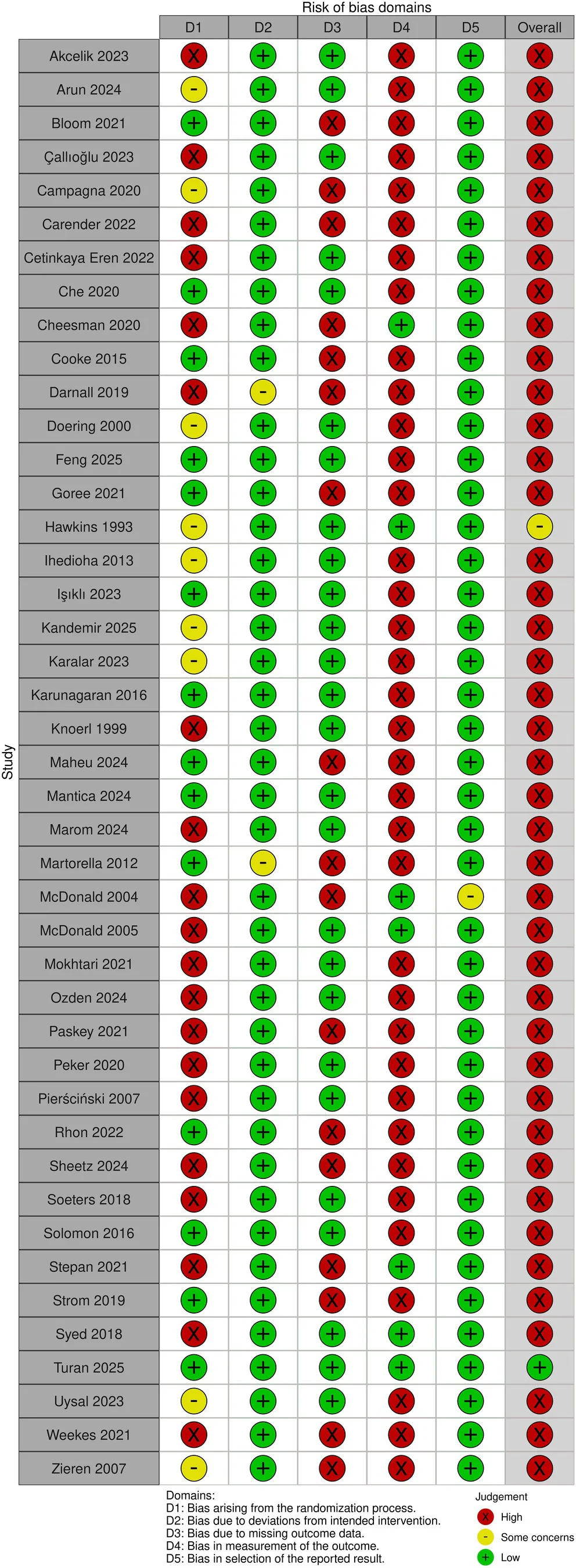
Risk of bias assessment regarding pain intensity according to the domain‐based Cochrane risk of bias tool RoB 2.

### 3.3 Primary Outcome: Postoperative Pain

Eleven of the 42 trials assessing pain found significantly lower pain levels in the video intervention group compared to the control group during the postoperative period (Table 2) [25, 27, 30, 40, 41, 47, 50, 51, 55, 58, 64]. Of the 11 trials with a significant difference, seven showed a difference greater than the 30% minimal clinically important difference threshold [27, 30, 47, 50, 51, 55, 58]. One of the included trials showed significantly higher pain levels in the intervention group compared to the control group [26], and the remaining 30 trials showed no significant difference between groups [24, 28, 29, 31, 33, 34, 35, 36, 37, 38, 39, 42, 43, 44, 45, 46, 48, 49, 52, 53, 54, 56, 57, 59, 60, 61, 63, 65, 67, 68].

**TABLE 2:** Main results: Primary and secondary outcomes.

First author (Year)	Postoperative pain intensity	Postoperative analgesic consumption	Differences in secondary outcomes
Akcelik (2023) [24]	No significant differences between groups	NA	Greater satisfaction in video and brochure group compared to the control Higher physical functioning at 6 weeks in video group compared to the control
Arun (2024) [25]	Lower pain at 12 h for the intervention group, values not presented, *p* < 0.04.	NA	Decrease in anxiety after receiving education for both groups
Bloom (2021) [26]	POD 4 VAS: 5.6 intervention group vs. VAS 4.8 control, *p* = 0.03 No significant differences between groups POD 1 or 7.	No significant differences between groups	No
Bozoghlian (2025) [62]	NA	No significant differences between groups	No
Çallıoğlu (2023) [27]	VAS: 3 intervention group vs. VAS 4.7 control group, *p* ≤ 0.001	NA	Shorter channel transition time in the video group
Campagna (2020) [28]	No significant differences between groups	NA	No
Carender (2022) [29]	No significant differences between groups	6 weeks postop best case OMEQs: 98 video + ACT vs. 330 control, p ≤ 0.001. A total of 195 OMEQs video vs. 330 control, *p* = 0.04. Six weeks postop worst case OMEQs: 500 video + ACT vs. 500 control, *p* = 0.04. No significant difference between groups at weeks best or worst case and 6 weeks worst case video vs. control.	Significantly shorter duration of opioid treatment in video + ACT and video compared to control Significantly fewer opioid refills in video + ACT vs. control
Cetinkaya Eren (2022) [30]	VAS at rest: POD 5 5 intervention group vs. 6.8 control group, *p* = 0.01. Three months postop 1.8 intervention group vs. VAS 3.5 control group, *p* = 0.01. VAS during activity: 3 months postop 3.5 intervention group vs. 5.5 control *p* = 0.01. No significant differences between groups during activity POD 5, sleep 5 days or 3 months postop and HHS pain at 3 months.	NA	Higher satisfaction for the intervention group Higher total score on the HHS and higher sub‐scores for function, absence of deformity and range‐of‐motion for intervention
Che (2020) [31]	No significant differences between groups	NA	Higher postop satisfaction in the intervention group Lower salivary cortisol in the intervention group after watching the video compared to the control group.
Cooke (2015) [63]	No significant differences between groups	NA	No
Darnall (2019) [33]	No significant differences between groups	NA	86% higher likelihood of opioid cessation within 12 weeks for the intervention group
Doering (2000) [34]	No significant differences between groups	POD 0–4 Lower consumption of opioids, *p* = 0.037. No absolute numbers presented.	Lower anxiety in the intervention group preoperatively and the first two days post‐op. More patients with intraoperative increase in systolic blood pressure in the control group. Lower cortisol excretion in urine preoperatively and the first 2 days post‐op in the intervention group. Lower consumption of nonsteroidal antirheumatics and total analgesics in the intervention group.
Feng (2025) [64]	SF‐36 Bodily Pain: 81.6 intervention group vs. 76.5 in control group, *p* < 0.05	NA	Higher satisfaction and knowledge in the intervention group Higher increase in social support pre‐ to postop in the intervention group Higher scores in all domains of quality of life according to SF‐36‐scores
Goree (2021) [35]	No significant differences between groups	NA	Higher knowledge of opioids postop in the video group
Hawkins (1993) [36]	No significant differences between groups	NA	95% of the patients who were shown the pain video recommended it to be available to all patients preoperatively. Patients expected better pain relief from medication after the pain video. Patients felt less awkward asking for pain medication in the pain video group Patients in the pain video group asked for pain medication at milder pain
Ihedioha (2013) [37]	No significant differences between groups	No significant differences between groups	No
Işıklı (2023) [38]	No significant differences between groups	NA	Lower level of fear for severe pain before the surgery in the intervention group Increase in pain pre‐ vs. postoperative in the control group
Kandemir (2025) [65]	No significant differences between groups	NA	Significantly lower postop STAI‐scores and stress in the intervention group.
Karalar (2023) [39]	No significant differences between groups	NA	Lower anxiety on APAIS and STAI‐state in the intervention group Higher willingness to do the surgery again in the intervention group
Karunagaran (2016) [40]	No pain: 72.2% in the intervention group vs. 47.2% control group. Mild pain: 25% in the intervention group vs. 52.3% control group. *p* = 0.04	NA	Decrease in anxiety after receiving education in the intervention group, not the control group
Knoerl (1999) [41]	Worst pain NRS: 6.5 intervention group vs. NRS 7.7 control group, *p* = 0.02 No significant differences between groups at 4, 8, 24 h or concerning least pain.	No significant differences between groups	Better knowledge and attitude regarding pain medicine after education Higher satisfaction 4 and 8 h post‐op in the intervention group
Lubitz (2024) [66]	NA	No significant differences between groups	No
Maheu (2024) [42]	No significant differences between groups	POD 3 OMEQs: 15.4 intervention group vs. 22.1 control group, *p* = 0,038. POD 4 OMEQs: 14 intervention group vs. 21.4 control group, *p* = 0.024. POD 0–3 OMEQs 78.8 intervention group vs. 106.1 control group, *p* = 0.020. POD 0–7 OMEQs: 129.9 intervention vs. 180.7 control group *p* = 0.028. No significant differences between groups POD 0–2 or 5–14 as well as combined day 0–14.	No
Mantica (2024) [43]	No significant differences between groups	NA	Lower anxiety on STAI after the information process in the video group Greater difference in STAI before/after the information process in the video group Higher satisfaction in the video group. Fewer calls for nurse in the video group
Marom (2024) [44]	No significant differences between groups	NA	Lower anxiety during the procedure in the video group Lower pain in the intervention group in the subanalysis of patients with first‐time amniocentesis Lower anxiety during the procedure in patients with first‐time amniocentesis
Martorella (2012) [45]	No significant differences between groups	POD 2 OMEQs: 31.2 intervention group vs. 18.8 control group, *p* = 0.006. No significant differences between groups POD 1, 3 or 7.	Lower pain interference with appetite in the intervention group postop day 7 Lower pain interference with breathing and coughing in the intervention group postop day 3 Fewer barriers related to harmful effects of analgesia in the intervention group
McDonald (2005) [46]	No significant differences between groups	NA	Lower pain interference in the intervention group postop day 1
McDonald (2004) [47]	POD 1: Lower sensory pain in the communication group compared to the pain management group, *p* < 0.02. No significant difference between groups in VAS POD 1 and POD 2, PPI POD 3 and POD 4, sensory pain POD 2–4, affective pain POD 1–4.	NA	No
Mokhtari (2021) [48]	No significant differences between groups	1 week postop oxycodone use: 1 intervention group vs. 7 in control, *p* < 0.001 2 weeks postop oxycodone use: 1.5 in intervention group vs. 10 in control group, *p* < 0.001	No
Ozden (2024) [49]	No significant differences between groups	NA	Higher pain during urodynamics in the control group Lower anxiety before urodynamics with brochure‐assisted video Lower satisfaction regarding education in the control and brochure groups
Paskey (2021) [50]	POD 3 VAS: 3.7 intervention group vs. VAS 4.9 control group, *p* = 0.04. POD 4 VAS: 3 intervention group vs. VAS 4.3 control group, *p* = 0.02. POD 5 VAS: 2.4 intervention group vs. VAS 3.5 control group, *p* = 0.04. No significant differences between groups POD 0, 1 or 2.	POD 5 OMEQs: 2 intervention group vs. 7.2 control, *p* = 0.01. Total OMEQs day 0–14: 46.4 intervention vs. 92.7 control, *p* = 0.02 No significant difference between groups POD 0–4.	No
Peker (2020) [51]	1 month post op SF‐36 Bodily Pain: 52 intervention group vs. 22 control group, *p* < 0.001 3 months post op SF‐36 Bodily Pain: 84 intervention group vs. 51 control group, *p* < 0.001	NA	All measured domains were better in the intervention group, including general health, mental health, physical function and vitality
Pierściński (2007) [52]	No significant differences between groups	No significant differences between groups	No
Rhon (2022) [53]	No significant differences between groups	No significant differences between groups	No
Sheetz (2024) [54]	No significant differences between groups	No significant differences between groups	No
Soeters (2018) [55]	WOMAC‐pain score: 1 intervention group vs. 3 control group, *p* ≤ 0.001	NA	Education group met criteria for discharge from physical therapist earlier Better postop function and less stiffness in education group
Solomon (2016) [56]	No significant differences between groups	NA	The music group felt less prepared than standard care and video
Stepan (2021) [57]	No significant differences between groups	Pills consumed 2 intervention group vs. 5 control group, *p* ≤ 0.001 OMEQ 7.8 intervention group vs. 40 control group, *p* ≤ 0.001 Percentage with no opioid consumption: 41.9% intervention group vs. 34.5% control group, *p* = 0.01	The intervention group took more NSAIDS The control group more refill prescriptions The control group more dissatisfied with their surgery
Strom (2019) [67]	No significant differences between groups	NA	No
Syed (2018) [58]	2 weeks postop VAS: 3.3 intervention group vs. 4.4 control, *p* = 0.008 6 weeks postop VAS: 2.4 intervention group vs. 3.7 control, *p* = 0.001 No significant differences between groups at 3 months or a minimum of 2 years.	6 weeks postop pills consumed: 40.4 intervention group vs. 60.6 control group, *p* = 0.02 3 months postop pills consumed: 51.2 intervention group vs. 87.2 control group, *p* = 0.01 No significant differences between groups at 2 weeks or at a minimum 2 years postop.	Preoperative narcotic use and VAS score at 3 months were associated with higher narcotic consumption at 3 months. Among patients taking narcotics preoperatively, the effect of education was higher. Lower number of prescription refills and opioid dependence in the intervention group at a minimum of 2 years
Turan (2025) [68]	No significant differences between groups	No significant differences between groups	No
Uysal (2023) [59]	No significant differences between groups	No significant differences between groups	No
Weekes (2021) [60]	No significant differences between groups	2 weeks post op OMEQs: 309 intervention group vs. 442 control group, *p* ≤ 0.01 No significant differences between groups POD 0–5.	No
Zieren (2007) [61]	No significant differences between groups	NA	Higher quality of life in the first 3 months postop in the intervention group. Higher values for social roles and global health postop in the intervention group. Shorter time to resume sport and professional activities after surgery in the intervention group.

Abbreviations: ACT, acceptance and commitment therapy; APAIS, Amsterdam preoperative anxiety and information scale; HHS, Harris Hip scale; NA, not assessed; NRS, numerical rating scale; NSAIDs, non‐steroidal anti‐inflammatory drugs; OMEQs, oral morphine equivalents; POD, postoperative day; SF‐36, short form (36) health survey; STAI, state–trait anxiety inventory; VAS, visual analog scale; WOMAC, The Western Ontario and McMaster Universities Arthritis Index.

### 3.4 Primary Outcome: Analgesic Consumption

Among the 19 trials that measured analgesic consumption, eight showed significantly lower consumption in the intervention group compared to the control group (Table 2) [29, 34, 42, 48, 50, 57, 58, 60]. Of these eight, six showed a reduction greater than 30% [42, 48, 50, 57, 58, 60]. One trial found significantly higher analgesia consumption in the intervention group compared to the control group [45]; the remaining 10 trials showed no significant difference between the groups [26, 37, 41, 52, 53, 54, 59, 62, 66, 68].

### 3.5 Combined Analysis of the Co‐Primary Outcomes

Only two of the 44 trials showed both significantly reduced pain intensity and analgesic consumption in the group receiving the video intervention [50, 58]. This contrasts with the eight trials that found no significant difference in either pain levels or analgesic consumption [35, 36, 37, 52, 53, 54, 59, 68]. Seventeen trials found a significant reduction in pain levels or analgesic consumption (including the two trials with significant results for both parameters) [25, 27, 29, 30, 34, 40, 41, 42, 47, 48, 50, 51, 55, 57, 58, 60, 64], while two trials found a significant increase in these measures [26, 45].

### 3.6 Certainty of Evidence

For both primary outcomes (postoperative pain and analgesic consumption) we assessed the certainty of evidence narratively. Most included trials were judged to be at high risk of bias, representing a serious concern. Results were inconsistent across trials (only 2 of 44 trials demonstrated benefit for both co‐primary outcomes). The evidence was direct and based on a large number of participants, and publication bias was considered unlikely given the mixed findings across published trials. Overall, the certainty of evidence was rated as low for both primary outcomes.

### 3.7 Other Outcomes

Twenty‐six of the 44 included trials reported significant improvements in outcomes other than the primary outcomes among participants who received the video intervention compared to controls [24, 25, 27, 30, 31, 33, 34, 35, 36, 38, 39, 40, 41, 43, 44, 45, 46, 49, 51, 55, 56, 57, 58, 61, 64, 65]. Beneficial effects were observed in measures such as pain interference, anxiety, satisfaction, physical function, likelihood of opioid cessation, and salivary cortisol levels (with lower cortisol levels indicating reduced stress response) (Table 2).

### 3.8 Subgroup Analyses

Table 3 presents an overview of subgroup analyses that investigate the effect of factors related to study design.

**TABLE 3:** Overview of results from subgroup analyses.

	Postoperative pain intensity			Postoperative analgesic consumption		
	Lower^a^	Higher^b^	No difference	Lower^a^	Higher^b^	No difference
All included studies	11/42	1/42	30/42	8/19	1/19	10/19
Video duration^c^						
Short	3/15	0/15	12/15	6/8	0/8	2/8
Medium	3/11	1/11	7/11	2/6	0/6	4/6
Long	0/5	0/5	5/5	0/1	0/1	1/1
Video showed by a researcher or healthcare worker	5/22	0/22	17/22	4/9	1/9	4/9
Video that could be watched anywhere	3/7	0/7	4/7	1/2	0/2	1/2
Video watched once	5/20	0/20	15/20	4/8	1/8	3/8
Possibility to rewatch video	3/10	0/10	7/10	1/5	0/5	4/5
Video shown/accessed preoperatively	9/39	1/39	29/39	7/17	1/17	9/17
Video shown/accessed postoperatively	2/3	0/3	1/3	1/2	0/2	1/2
Mention of professional help in making video	3/12	0/12	9/12	1/3	1/3	1/3
No mention of professional help in making video	8/30	1/30	21/30	7/16	0/16	9/16
Pain intensity and/or analgesic consumption primary outcome	6/26	1/26	19/26	7/17	1/17	9/17
Pain intensity and/or analgesic consumption not the primary outcome	5/16	0/16	11/16	1/2	0/2	1/2
Video standalone intervention	4/23	1/23	18/23	4/9	0/9	5/9
Video part of the intervention	7/19	0/19	11/19	4/10	1/10	5/10
Major surgery	9/34	1/34	24/34	8/19	1/19	10/19
Minor surgery	2/8	0/8	6/8	—	—	—
Pain‐ or analgesia‐related exclusion criteria	5/20	1/20	14/20	7/16	1/16	8/16
No pain‐ or analgesia‐related exclusion criteria	6/22	0/22	16/22	1/3	0/3	2/3
Main video content^d^						
Education	8/25	1/25	16/25	6/15	1/15	8/15
Information	3/17	0/17	14/17	2/4	0/4	2/4
Distribution in terms of sex						
100% female sample	2/9	0/9	7/9	1/2	0/2	1/2
> 50% female sample	7/23	0/23	16/23	3/6	0/6	3/6
Equal distribution	0/1	0/1	1/1	—	—	—
< 50% female sample	4/16	1/16	11/16	5/13	1/13	7/13
Sample size						
> More than 100 participants	5/15	1/15	9/15	5/10	0/10	5/10
≤ 100 participants	6/27	0/27	21/27	3/9	1/9	5/9
Timing of postoperative outcome assessment^e^						
Acute	8/37	1/37	28/37	4/16	1/16	11/16
Subacute	1/11	0/11	10/11	4/10	0/10	6/10
Long‐term	4/11	0/11	7/11	2/5	0/5	3/5
Risk of bias						
Low	0/2	0/2	2/2	0/2	0/2	2/2
Some concerns	0/1	0/1	1/1	—	—	—
High	11/39	1/39	27/39	8/17	1/17	8/17

^a^ Significantly lower values in the intervention group compared to the control group.

^b^ Significantly higher values in the intervention group compared to the control group.

^c^ Short 0–6 min, medium 6–16 min and long ≥ 16 min duration.

^d^ Educational videos gave participants new tools, while informational videos described upcoming events.

^e^ Acute phase first 7 days, subacute week 1–4 and long‐term > 4 weeks postop.

#### 3.8.1 Trials With Beneficial Outcomes for Both Pain Intensity and Analgesic Consumption

Two trials demonstrated statistically significant improvements in both postoperative pain intensity and analgesic consumption [50, 58]. These two trials shared several key characteristics: both designated pain or analgesia consumption as the primary outcome, applied pain‐related exclusion criteria, included subacute measurements, involved major surgeries, and utilized short educational videos.

## 4 Discussion

The findings of the current systematic review suggest that reductions in postoperative pain and/or analgesic consumption may be achievable with the use of a video intervention across a broad range of surgical procedures. Among trials reporting a statistically significant reduction in pain, most also exceeded the predefined 30% MCID threshold, suggesting the effects were potentially clinically meaningful rather than merely statistically detectable. This distinction is important, as statistical significance does not guarantee that a difference is perceptible to patients or sufficient to alter clinical practice. However, these findings should be interpreted in light of the high risk of bias identified in most trials and the low certainty of the evidence. In comparison, a 2020 systematic review by Sorel et al. which focused specifically on total knee arthroplasty, examined various perioperative interventions for psychological distress and found that 20 of 34 trials reported improved pain scores or reduced postoperative opioid prescriptions [69]. Despite the wider scope of interventions analyzed, their review similarly indicates that beneficial effects are observed only in select cases.

### 4.1 Context‐Dependent Efficacy

Given the wide range of surgical contexts, from minor procedures (e.g., office hysteroscopy, gastroscopy) to major abdominal and thoracic surgery, each with vastly different pain trajectories, pooling results is problematic. Nevertheless, several trials demonstrate significant beneficial effects on pain control. For example, Hirschey et al. examined the effects of video education in underrepresented minority cancer survivors and found that video education could help reduce pain in this patient category [70]. Similarly, while Marom et al. reported no overall group difference, a subgroup of first‐time amniocentesis patients experienced significantly lower procedural pain in the video group than in the control group [44]. These findings support the conclusion that video interventions are effective in select cases. This raises an important question: does efficacy arise because certain patient groups have a greater need for preparatory education, because certain video designs are better suited to particular contexts, or due to differences in the quality of video interventions?

### 4.2 The Pain‐Analgesia Relationship

When interpreting these results, it is important to consider the relationship between pain and analgesic consumption. While it seems intuitive that higher pain levels would drive increased analgesic consumption, it is equally possible that higher consumption is the cause of the lower reported pain scores. Therefore, any trial that demonstrates improved postoperative pain control, whether by reducing analgesia consumption without increasing pain levels or by lowering pain levels without increasing analgesia consumption, should be considered successful. The fact that many trials only examined pain levels, not differences in analgesic consumption, is a significant limitation; without this data, we cannot fully understand the effectiveness of the video intervention. A notable example of a comprehensive approach is the trial by Doering et al. which analyzed opioid consumption, nonsteroidal anti‐inflammatory drug use, and total analgesic consumption, providing greater depth to its findings [34]. In light of this discussion, the higher frequency of trials reporting a significant effect on analgesic outcomes compared to pain outcomes in the present review is noteworthy. In contrast with our results, and further underscoring the complex pain‐analgesia relationship, a systematic review by Szeverenyi et al. on adjunct psychosocial interventions reported significantly lower pain levels but no significant reduction in analgesic consumption [71].

### 4.3 Trials Reporting Higher Pain Scores or Analgesic Consumption

Notably, the trial by Bloom et al. which aimed to reduce postoperative opioid consumption, found higher pain scores in the intervention group only on postoperative day (POD) 4; scores on POD 1 and 7 were similar between the groups [26]. This outcome might be influenced by the video intervention itself, which reportedly discusses the risks of narcotic abuse, potentially causing patients to refrain from opioid use despite experiencing significant pain. It should also be noted that the trial by Martorella et al. which reported higher analgesic consumption in the intervention group, included information regarding timely pain relief and the promotion of pain self‐management in the intervention [45].

### 4.4 The Critical Role of Participant Engagement

The effectiveness of a video intervention obviously depends on participant engagement, as illustrated by Lubitz et al. Among 89 participants given access to a website containing the intervention, only approximately 30% visited the site, and the video was viewed just five times in total [66]. Consequently, their finding of no significant difference between trial groups is unsurprising. To mitigate this engagement barrier, many trials in this systematic review administered the video intervention in a hospital setting. While this ensures exposure, it does not guarantee deep engagement or commitment, which, especially after discharge from the hospital, is essential for a successful intervention. This engagement challenge may partially explain why interventions where the video was part of a bundled approach, rather than standalone, showed a higher frequency of success. To properly evaluate the effect of video interventions, more trials should report data on patient engagement, for example, the frequency and timing of video views. Furthermore, methods to increase participant engagement should be a major priority for future research. Although not via video interventions, Enhanced Recovery After Surgery (ERAS) protocols have been shown to decrease analgesic consumption through non‐pharmacological treatment options and education of patients and their families, among other strategies [13]. This further supports the possibility that video interventions can have positive effects, provided the intervention is of sufficient quality and patients are adequately engaged.

### 4.5 Implications for Future Research

As noted in the introduction, a well‐designed video intervention offers significant benefits for the individual patient and represents a cost‐effective strategy for healthcare systems [18, 19]. This systematic review, consistent with other recent reviews [69], concludes that perioperative video interventions may demonstrate efficacy in select cases. Therefore, future research should prioritize identifying the specific factors that correlate with intervention success. In the present review, several characteristics were associated with a higher frequency of positive outcomes and warrant further investigation, for example, remote accessibility, integration into a multimodal intervention (rather than standalone use), and an educational focus.

### 4.6 Limitations

Vast methodological heterogeneity exists among the included trials, including differences in video length and in the timing of interventions and/or outcome measurements. The wide range of surgical contexts, spanning minor procedures such as gastroscopy, where low pain scores and negligible opioid use are expected, to major abdominal and thoracic surgery with entirely different pain trajectories, renders direct comparison of findings and meta‐analysis inappropriate. Hence, this review had to rely solely on a comprehensive qualitative appraisal. Because video interventions varied widely in content (from procedural explanations to pain management techniques), the lack of consistency complicates a focused evaluation of the intervention's specific impact. Comparability between trials was further limited by the fact that some interventions were within a package of care involving psychological components. While the broad inclusion period (1993–2025) introduces potential confounding factors due to technological advancements in video interventions, it was necessary to capture the full scope of research for a comprehensive systematic review. The common practice of measuring outcomes at multiple time points meant that in some cases a significant difference was identified at only a single time point. As previously discussed, many trials assessed only one of the two co‐primary outcome measures; consequently, a non‐significant finding may obscure a meaningful effect in the unmeasured outcome. Analgesic co‐interventions were permitted in the included trials, but reporting of their use was inconsistent, and few studies reported whether use was balanced between arms; this limits our ability to assess potential confounding by differential analgesic use.

An additional limitation is that 16 trials examining pain and two trials measuring analgesic consumption defined a primary outcome measure other than those specified in this review. Moreover, almost all trials were judged to have a high risk of bias, which could have influenced the results. The certainty of evidence was not formally assessed using the GRADE approach, as heterogeneity of outcome measures precluded pooling, and results were inconsistent across trials. Finally, while it is possible that not all relevant trials were identified, this risk is considered minimal as the reference lists of all included full‐text articles were screened.

## 5 Conclusion

Perioperative video interventions may provide benefits in reducing postoperative pain intensity and analgesic consumption, although the evidence is of low certainty and their efficacy appears inconsistent across trials. This inconsistency may be contingent on factors such as video duration, content focus, delivery setting, and participant engagement. Future research is needed to identify the patient profiles and intervention characteristics that predict success, to enable the development of targeted, evidence‐based programs for clinical implementation.

## Author Contributions

**A.D.:** conceptualization, methodology, investigation, data analysis, writing – original draft; **S.A.:** methodology, investigation, data analysis, writing – review and editing; **A.F.:** investigation, writing – review and editing; **M.L.:** conceptualization, methodology, writing – review and editing; **E.K.:** conceptualization, methodology, writing – review and editing; **M.F.B.:** conceptualization, methodology, investigation, data analysis, writing – original draft, supervision.

## Funding

This work was funded by the Acta Anaesthesiologica Scandinavica Foundation; Swedish Government (national and regional ALF grants).

## Ethics Statement

The authors have nothing to report.

## Consent

The authors have nothing to report.

## Conflicts of Interest

The authors declare no conflicts of interest.

## Supporting information


**Data S1:** Supporting Information.

**Supplemental Figure 1:** Risk of bias assessment regarding analgesic consumption according to the domain‐based Cochrane risk of bias tool RoB 2.

**Supplemental Table 1:** Additional data from the included studies.

**Supplemental Table 2:** Risk of Bias Assessment regarding pain intensity.

**Supplemental Table 3:** Risk of Bias Assessment regarding analgesic consumption.

## Data Availability

All data generated or analysed during this study are included in the published article and its Supporting Information files.
