# Supplementary material for: The Effect of Perioperative Video Interventions on Postoperative Pain Control: A Systematic Review
Source: Acta Anaesthesiol Scand. 2026 Nov 1;71(1):e70387. doi: 10.1111/aas.70387 (PMC13724283; doi:10.1111/aas.70387)
Supplement: Supplementary file 1 [file AAS-71-0-s001.docx]

# Supplemental Document 1 – Database Search Strategy

**PubMed 2024-10-04**

**#1**

"Videotape Recording"[Mesh] OR video*[Title/Abstract] OR video-based[Title/Abstract] OR video-assisted[Title/Abstract]

190,718

**#2**

"Pain, Postoperative"[Mesh] OR "Pain Measurement"[Mesh] OR ("pain management"[Title/Abstract:~3]) OR ("Perioperative Care"[Mesh]) OR ("Preoperative Care"[Mesh]) OR "pain level"[Title/Abstract:~3] OR "pain levels"[Title/Abstract:~3] OR "pain management"[Title/Abstract:~3] OR "perioperative counseling"[Title/Abstract:~3] OR "postoperative counseling"[Title/Abstract:~3] OR "perioperative education"[Title/Abstract:~3] OR "postoperative education"[Title/Abstract:~3]

346,265

**#3**

"patient education"[Title/Abstract:~3] OR "patient recovery"[Title/Abstract:~3] OR "patient outcome"[Title/Abstract:~3] OR "patient intervention"[Title/Abstract:~3] OR "patient outcomes"[Title/Abstract:~3] OR "patient interventions"[Title/Abstract:~3] OR "patient satisfaction"[Title/Abstract:~3] OR "patient stress"[Title/Abstract:~3] OR "patient anxiety"[Title/Abstract:~3] OR "patients outcome"[Title/Abstract:~3] OR "patients interventions"[Title/Abstract:~3] OR "patients fear"[Title/Abstract:~3] OR "patients stress"[Title/Abstract:~3] OR "patients fear"[Title/Abstract:~3] OR "Patient Education as Topic"[Mesh] OR "Patient Satisfaction"[Mesh] OR "fear pain"[Title/Abstract:~3] OR "pain control"[Title/Abstract:~3] OR "opioid education"[Title/Abstract:~3] OR "opioid prescribing"[Title/Abstract:~3] OR "opioid knowledge"[Title/Abstract:~3] OR "opioid counseling"[Title/Abstract:~3]

661,001

**#4**

#1 AND #2 AND #3 AND English

846

**Embase 2024-10-04**

**#1**

video* NOT (‘video consultation’ OR ‘video consultations’ OR teleconsultation)

299,198

**#2**

'postoperative pain'/exp OR 'pain assessment':ti,ab OR 'pain management':ti,ab OR 'pain control':ti,ab OR 'preoperative education':ti,ab OR 'opioid education':ti,ab OR 'pain control':ti,ab OR 'pain control':ti,ab OR 'pain perception':ti,ab

171,592

**#3**

'patient satisfaction'/exp OR 'patient education'/exp OR 'patient education':ti,ab OR 'patient satisfaction':ti,ab OR preoperative NEAR/3 counseling OR anxiety:ti,ab OR fear NEAR/2 pain OR self-care NEAR/2 knowledge OR opioid NEAR/3 knowledge OR patient* NEAR/2 experience* OR 'preoperative evaluation' OR 'postoperative evaluation' OR preoperative NEAR/3 counseling OR 'preoperative assessment' OR 'postoperative assessment' OR preoperative NEAR/2 education

1,118,502

**#4**

#1 AND #2 AND #3

883

**#5**

#4 AND ('article'/it OR 'article in press'/it OR 'review'/it) AND [english]/lim

634

**Cinahl 2024-10-04**

**#1**

(MH "Videorecording+") OR video*

77,629

**#2**

(MH "Postoperative Pain") OR MH "Pain Measurement" OR (pain N3 (assessment OR management OR control OR education OR perception)) OR (preoperative N3 education) OR (opioid N3 education) OR (MM "Preoperative Care") OR "surgical patients" N3 education

131,727

**#3**

(MH "Patient Satisfaction+") OR (MM "Preoperative Education") OR (MM "Patient Education") OR (MM "Health Education") OR patient N3 (satisfaction OR education OR anxiety OR stress) OR preoperative N3 (counseling OR education OR evaluation OR assessment) OR anxiety N3 control OR self-care N3 (knowledge OR education)

217,643

**#4**

#1 AND #2 AND #3

431

**#5**

#4 AND academic journals, english

388

**Academic Search Complete 2024-10-04**

**#1**

DE "VIDEO recording" OR TI video* OR AB video*

338,704

**#2**

DE "POSTOPERATIVE pain" OR DE "PAIN measurement" OR (pain N3 (assessment OR management OR control OR education OR perception OR surgery)) OR (preoperative N3 education) OR (opioid N3 education)

115,121

**#3**

DE "PATIENT satisfaction" OR "Preoperative Education") OR "Patient Education" OR "Health Education") OR “activities of daily living” OR patient N2 (satisfaction OR education OR anxiety OR stress) OR preoperative N3 (counseling OR education OR evaluation OR assessment) OR self-care N3 (knowledge OR education)

212,991

**#4**

#1 AND #2 AND #3

225

**#5**

#4 AND academic journals, english

221

**Cochrane Database of Systematic Reviews 2024-10-14**

**#1**	(video*):ti,ab,kw	36137

**#2**	MeSH descriptor: [Pain, Postoperative] explode all trees	22000

**#3**	((Pain Measurement) OR (pain management) OR (Perioperative Care) OR (Preoperative Care) OR (pain management) OR (perioperative counseling) OR (postoperative counseling) OR (perioperative education) OR (postoperative education)):ti,ab,kw	149149

**#4**	#2 OR #3	157515

**#5**	("patient education" OR "patient recovery" OR "patient outcome" OR "patient intervention" OR "patient outcomes" OR "patient interventions" OR "patient satisfaction" OR "patient stress" OR "patient anxiety" OR "patients outcome" OR "patients interventions" OR "patients fear" OR "patients stress" OR "patients fear" OR "Patient Satisfaction" OR "pain control"):ti,ab,kw	76397

**#6**	#1 AND #4 AND #5 AND Cochrane Reviews	10

**PubMed 2025-10-16**

**#1**

"Videotape Recording"[Mesh] OR video*[Title/Abstract] OR video-based[Title/Abstract] OR video-assisted[Title/Abstract]

206,400

**#2**

"Pain, Postoperative"[Mesh] OR "Pain Measurement"[Mesh] OR ("pain management"[Title/Abstract:~3]) OR ("Perioperative Care"[Mesh]) OR ("Preoperative Care"[Mesh]) OR "pain level"[Title/Abstract:~3] OR "pain levels"[Title/Abstract:~3] OR "pain management"[Title/Abstract:~3] OR "perioperative counseling"[Title/Abstract:~3] OR "postoperative counseling"[Title/Abstract:~3] OR "perioperative education"[Title/Abstract:~3] OR "postoperative education"[Title/Abstract:~3]

363,763

**#3**

"patient education"[Title/Abstract:~3] OR "patient recovery"[Title/Abstract:~3] OR "patient outcome"[Title/Abstract:~3] OR "patient intervention"[Title/Abstract:~3] OR "patient outcomes"[Title/Abstract:~3] OR "patient interventions"[Title/Abstract:~3] OR "patient satisfaction"[Title/Abstract:~3] OR "patient stress"[Title/Abstract:~3] OR "patient anxiety"[Title/Abstract:~3] OR "patients outcome"[Title/Abstract:~3] OR "patients interventions"[Title/Abstract:~3] OR "patients fear"[Title/Abstract:~3] OR "patients stress"[Title/Abstract:~3] OR "patients fear"[Title/Abstract:~3] OR "Patient Education as Topic"[Mesh] OR "Patient Satisfaction"[Mesh] OR "fear pain"[Title/Abstract:~3] OR "pain control"[Title/Abstract:~3] OR "opioid education"[Title/Abstract:~3] OR "opioid prescribing"[Title/Abstract:~3] OR "opioid knowledge"[Title/Abstract:~3] OR "opioid counseling"[Title/Abstract:~3]

725,928

**#4**

#1 AND #2 AND #3 AND English

986

**#5**

#4 AND PY: 2024-10-01-2025-10-30

144

**Embase 2025-10-16**

**#1**

video* NOT (‘video consultation’ OR ‘video consultations’ OR teleconsultation)

337,723

**#2**

'postoperative pain'/exp OR 'pain assessment':ti,ab OR 'pain management':ti,ab OR 'pain control':ti,ab OR 'preoperative education':ti,ab OR 'opioid education':ti,ab OR 'pain control':ti,ab OR 'pain control':ti,ab OR 'pain perception':ti,ab

204,221

**#3**

'patient satisfaction'/exp OR 'patient education'/exp OR 'patient education':ti,ab OR 'patient satisfaction':ti,ab OR preoperative NEAR/3 counseling OR anxiety:ti,ab OR fear NEAR/2 pain OR self-care NEAR/2 knowledge OR opioid NEAR/3 knowledge OR patient* NEAR/2 experience* OR 'preoperative evaluation' OR 'postoperative evaluation' OR preoperative NEAR/3 counseling OR 'preoperative assessment' OR 'postoperative assessment' OR preoperative NEAR/2 education

1,244,898

**#4**

#1 AND #2 AND #3

1,349

**#5**

#4 AND ('article'/it OR 'article in press'/it OR 'review'/it) AND [english]/lim

733

**#6**

#5 AND PY: 2024-10-01-2025-10-30

168

**Cinahl 2025-10-16**

**#1**

(MH "Videorecording+") OR video*

82,468

**#2**

(MH "Postoperative Pain") OR MH "Pain Measurement" OR (pain N3 (assessment OR management OR control OR education OR perception)) OR (preoperative N3 education) OR (opioid N3 education) OR (MM "Preoperative Care") OR "surgical patients" N3 education

139,099

**#3**

(MH "Patient Satisfaction+") OR (MM "Preoperative Education") OR (MM "Patient Education") OR (MM "Health Education") OR patient N3 (satisfaction OR education OR anxiety OR stress) OR preoperative N3 (counseling OR education OR evaluation OR assessment) OR anxiety N3 control OR self-care N3 (knowledge OR education)

229,291

**#4**

#1 AND #2 AND #3

483

**#5**

#4 AND academic journals, English

437

**#6**

#5 AND PY: 2024-10-01-2025-10-30

48

**Academic Search Complete 2025-10-16**

**#1**

DE "VIDEO recording" OR XB (video)

321,162

**#2**

DE "POSTOPERATIVE pain" OR DE "PAIN measurement" OR (pain N3 (assessment OR management OR control OR education OR perception OR surgery)) OR (preoperative N3 education) OR (opioid N3 education)

126,045

**#3**

DE "PATIENT satisfaction" OR "Preoperative Education") OR "Patient Education" OR "Health Education") OR “activities of daily living” OR patient N2 (satisfaction OR education OR anxiety OR stress) OR preoperative N3 (counseling OR education OR evaluation OR assessment) OR self-care N3 (knowledge OR education)

234,975

**#4**

#1 AND #2 AND #3

243

**#5**

#4 AND academic journals, English

237

**#6**

#5 AND PY: 2024-10-01-2025-10-30

37

**Cochrane Database of Systematic Reviews 2025-10-16**

**#1**	(video*):ti,ab,kw	39541

**#2**	MeSH descriptor: [Pain, Postoperative] explode all trees	22410

**#3**	((Pain Measurement) OR (pain management) OR (Perioperative Care) OR (Preoperative Care) OR (pain management) OR (perioperative counseling) OR (postoperative counseling) OR (perioperative education) OR (postoperative education)):ti,ab,kw	158979

**#4**	#2 OR #3	167397

**#5**	("patient education" OR "patient recovery" OR "patient outcome" OR "patient intervention" OR "patient outcomes" OR "patient interventions" OR "patient satisfaction" OR "patient stress" OR "patient anxiety" OR "patients outcome" OR "patients interventions" OR "patients fear" OR "patients stress" OR "patients fear" OR "Patient Satisfaction" OR "pain control"):ti,ab,kw	80867

**#6**	#1 AND #4 AND #5 AND Cochrane Reviews	10

**#7**	#6 AND PY: 2024-10-01-2025-10-30	0
